# Characterization and Management of Stable Coronary Artery Disease in Patients Undergoing Transcatheter Aortic Valve Implantation

**DOI:** 10.3390/jcm13123497

**Published:** 2024-06-14

**Authors:** Sofia Sammartino, Giulia Laterra, Thomas Pilgrim, Ignacio J. Amat Santos, Ole De Backer, Won-Keun Kim, Henrique Barbosa Ribeiro, Francesco Saia, Matjaz Bunc, Didier Tchetche, Philippe Garot, Flavio Luciano Ribichini, Darren Mylotte, Francesco Burzotta, Yusuke Watanabe, Francesco Bedogni, Tullio Tesorio, Tobias Rheude, Gennaro Sardella, Marco Tocci, Anna Franzone, Roberto Valvo, Mikko Savontaus, Hendrik Wienemann, Italo Porto, Caterina Gandolfo, Alessandro Iadanza, Alessandro Santo Bortone, Markus Mach, Azeem Latib, Luigi Biasco, Maurizio Taramasso, Federico De Marco, Valentina Frittitta, Elena Dipietro, Claudia Reddavid, Orazio Strazzieri, Silvia Motta, Alessandro Comis, Chiara Melfa, Mariachiara Calì, Carmelo Sgroi, Mohamed Abdel-Wahab, Giulio Stefanini, Corrado Tamburino, Marco Barbanti, Giuliano Costa

**Affiliations:** 1 Department of Cardiology, University of Catania, 95124 Catania, Italy; sofia.sammartino1@gmail.com (S.S.); frittittavalentina@gmail.com (V.F.); elenadipietro.ed@gmail.com (E.D.); claudiareddavid@gmail.com (C.R.); o.strazzieri@gmail.com (O.S.); silviamotta89@gmail.com (S.M.); alex.comis92@gmail.com (A.C.); chiara.melfa@gmail.com (C.M.); mariachiaracali@outlook.it (M.C.); giulianocosta90@gmail.com (G.C.); 2 Ospedale Umberto I, ASP 4 di Enna, 94100 Enna, Italy; giulia.laterra1990@gmail.com; 3 Bern University Hospital, Inselspital, 3010 Bern, Switzerland; thomas.pilgrim@insel.ch; 4 Division of Cardiology, Hospital Clínico Universitario de Valladolid, 47003 Valladolid, Spain; ijamat@gmail.com; 5 The Heart Center, Rigshospitalet, Copenhagen University Hospital, 1165 Copenhagen, Denmark; ole.de.backer@regionh.dk; 6 Kerckhoff Heart Center, 61231 Bad Nauheim, Germany; w.kim@kerckhoff-klinik.de; 7 Heart Institute of Sao Paulo (InCor), University of Sao Paulo, Sao Paulo 17012-901, Brazil; henriquebribeiro@me.com; 8 Cardiology Unit, Cardiac Thoracic and Vascular Department, IRCCS Azienda Ospedaliero-Universitaria di Bologna, 40138 Bologna, Italy; francescosaia@hotmail.com; 9 Department of Cardiology, University Medical Centre Ljubljana, 1525 Ljubljana, Slovenia; mbuncek@yahoo.com; 10 Clinique Pasteur, 31300 Toulouse, France; dtchetche@clinique-pasteur.com; 11 Institut Cardiovasculaire Paris-Sud, Hôpital Jacques Cartier, Ramsay-Santé, 91300 Massy, France; pgarot@angio-icps.com; 12 Division of Cardiology, Azienda Ospedaliera Universitaria Integrata di Verona, 37126 Verona, Italy; flavio.ribichini@univr.it; 13 Galway University Hospital, H91 YR71 Galway, Ireland; darrenmylotte@gmail.com; 14 Department of Cardiology, IRCSS Policlinico Universitario “Agostino Gemelli”, Università Cattolica del Sacro Cuore, 00168 Roma, Italy; francescoburzotta@gmail.com; 15 Department of Cardiology, Teikyo University School of Medicine, Tokyo 173-8605, Japan; yusuke0831@gmail.com; 16 Division of Cardiology, IRCSS Policlinico San Donato, 20097 San Donato Milanese, Italy; francescobedogni@tin.it (F.B.); roberto_valvo@hotmail.it (R.V.); 17 Clinica Montevergine, GVM Care & Research, 48033 Mercogliano, Italy; tulliotesorio@gmail.com; 18 German Heart Centre, 06120 Munich, Germany; tobias.rheude@googlemail.com; 19 Division of Cardiology, Policlinico Umberto I, 00185 Roma, Italy; rino.sardella@uniroma1.it (G.S.); marcotocci1392@gmail.com (M.T.); 20 Division of Cardiology, AOU Federico II, Università di Napoli, 80125 Napoli, Italy; anna.franzone@unina.it; 21 Heart Center, Turku University Hospital, 20014 Turku, Finland; mikko.savontaus@tyks.fi; 22 Faculty of Medicine, University Hospital Cologne, Clinic III for Internal Medicine, University of Cologne, 50923 Cologne, Germany; wienemann@uk-koeln.de; 23 CardioThoracic and Vascular Department, San Martino Policlinico Hospital, 16132 Genova, Italy; italo.porto@gmail.com; 24 Istituto Mediterraneo per i Trapianti e Terapie ad Alta Specializzazione (ISMETT), 90127 Palermo, Italy; caterinamariagandolfo@gmail.com; 25 Azienda Ospedaliera Universitaria Senese, UOSA Cardiologia Interventistica, Policlinico Le Scotte, 53100 Siena, Italy; alessandro.iadanza@gmail.com; 26 Division of University Cardiology, Cardiothoracic Department, Policlinico University Hospital, 70124 Bari, Italy; alessandrosanto.bortone@uniba.it; 27 Department of Cardiology, Medical University of Vienna, 1090 Vienna, Austria; markus.mach@meduniwien.ac.at; 28 Montefiore Medical Center, New York, NY 10461, USA; alatib@gmail.com; 29 Azienda Sanitaria Locale di Ciriè, Chivasso e Ivrea, ASLTO4, 10034 Chivasso, Italy; luigi.biasco@gmail.com; 30 Heart and Valve Center, University Hospital of Zurich, University of Zurich, 8006 Zurich, Switzerland; m.taramasso@gmail.com; 31 Centro Cardiologico Monzino, 20138 Milano, Italy; federico.demarco@gmail.com; 32 Division of Cardiology, A.O.U. Policlinico “G. Rodolico-San Marco”, 95123 Catania, Italy; carmelo_sgroi@hotmail.com (C.S.); tambucor@unict.it (C.T.); 33 Heart Center Leipzig, University of Leipzig, 04109 Leipzig, Germany; msabdelwahab@gmail.com; 34 IRCCS Istituto Clinico Humanitas, 20089 Rozzano, Italy; giulio.stefanini@gmail.com; 35 Department of Cardiology, Università degli Studi di Enna “Kore”, Piazza dell’Università, 94100 Enna, Italy

**Keywords:** transcatheter aortic valve implantation, coronary artery disease, percutaneous coronary intervention

## Abstract

**Background/Objectives**: To date, data regarding the characteristics and management of obstructive, stable coronary artery disease (CAD) encountered in patients undergoing transcatheter aortic valve implantation (TAVI) are sparse. The aim of the study was to analyze granular details, treatment, and outcomes of patients undergoing TAVI with obstructive, stable CAD from real-world practice. **Methods**: REVASC-TAVI (Management of myocardial REVASCularization in patients undergoing Transcatheter Aortic Valve Implantation with coronary artery disease) is an investigator-initiated, multicenter registry, which collected data from patients undergoing TAVI with obstructive stable CAD found during the pre-TAVI work-up. **Results**: A total of 2025 patients from 30 centers worldwide with complete follow-up were included in the registry. Most patients had single-vessel CAD (56.1%). An involvement of proximal coronary tracts was detected in 62.5% of cases, with 12.0% of patients having CAD in left main (LM). Most patients received percutaneous coronary intervention (PCI) (n = 1617, 79.9%), especially those with proximal CAD (90.4%). At 2 years, the rates of all-cause death [Kaplan–Meier (KM) estimates 20.1% vs. 18.8%, p_log-rank_ = 0.86] and of the composite of all-cause death, stroke, myocardial infarction, and rehospitalization for heart failure (KM estimates 29.7% vs. 27.5%, p_log-rank_ = 0.82) did not differ between patients undergoing PCI and those who were not. **Conclusions**: Patients undergoing TAVI with obstructive CAD more commonly had a single-vessel disease and an involvement of proximal coronary tracts. They were commonly treated with PCI, with similar outcomes compared to those treated conservatively.

## 1. Introduction

Transcatheter aortic valve implantation (TAVI) has become the treatment of choice for elderly patients affected by symptomatic severe aortic stenosis (AS) or for younger patients at increased surgical risk. Coronary artery disease (CAD) often coexists in patients affected by AS and undergoing TAVI [1]. Recently, the issue of coronary re-access after TAVI raised concern about the optimal management of obstructive, stable CAD, especially in patients with longer life expectancy. The European and American guidelines for valve heart diseases recommend to consider percutaneous coronary intervention (PCI) in patients undergoing TAVI with an involvement of proximal coronary vessels, but this statement is mainly based on expert consensus as existing data are poor [2,3]. Results from the recent ACTIVATION randomized clinical trial (RCT) did not show a clinical benefit of performing percutaneous coronary intervention (PCI) in TAVI recipients [4]. Notably, the results of this trial cannot be generalized to the current practice, due to the exclusion of patients with angina at baseline or unprotected left main (LM) disease. Characteristics of obstructive, stable CAD in patients undergoing TAVI in the real-world practice have been poorly investigated, as have its management and the clinical outcomes. The aim of the study was to report CAD details and clinical outcomes of TAVI patients with stable obstructive CAD treated in the current clinical practice and enrolled in the multicenter REVASC-TAVI registry, focusing on patients’ management in a real-world setting.

## 2. Materials and Methods

REVASC-TAVI (Management of myocardial REVASCularization in patients undergoing Transcatheter Aortic Valve Implantation with coronary artery disease) is an investigator-initiated, retrospective, multicenter registry, which enrolled patients undergoing TAVI with obstructive CAD, found at the coronary angiogram performed during the pre-TAVI work-up, from 30 institutions worldwide. Patients with missing follow-up and coronary angiogram data were excluded from the study. Baseline and clinical characteristics, echocardiographic parameters, coronary angiogram, TAVI and PCI specifics, and follow-up data were collected by the co-investigators at each center. Any discrepancies were resolved through direct communication with the respective local investigators. The management of CAD, as well as the indication for PCI, the utilization of functional invasive or noninvasive tests for myocardial ischemia and intravascular ultrasound (IVUS), PCI strategy, and the duration of antiplatelet therapy were subject to the discretion of each local heart team and operating interventional cardiologist.

All outcomes were defined according to the Valve Academic Research Consortium-2 (VARC-2) definitions [5].

Obstructive CAD was defined as the presence of visual angiographic stenosis ≥ 70% [≥50% if protected LM or vein graft], instantaneous wave-free ratio (iFR) value ≤ 0.89, fractional flow reserve (FFR) value ≤ 0.80, in 1 or more coronary arteries of at least 2.5 mm of diameter, not revascularized by patent coronary stents or bypass grafts, or LM minimal lumen area (MLA) < 6 mm^2^ at IVUS assessment. Coronary disease burden was assessed using the British Cardiovascular Intervention Society myocardial Jeopardy Score (BCIS-JS) [6].

The aim of the analysis is to report characteristics of CAD and details of the PCIs performed, rates of all-cause death and of the composite of all-cause death, stroke, myocardial infarction (MI), and rehospitalization for heart failure (HF) at 2 years, according to the strategy adopted for CAD management.

Categorical variables are reported as counts and percentages. Continuous variables are reported as medians and interquartile ranges (IQRs). Continuous variables were assessed using the t-test or Mann–Whitney U test, while categorical variables were evaluated using the chi-square statistics or Fischer’s exact test, as appropriate. Multivariable regression analyses were performed using generalized linear models. Results are reported as odds ratio (OR) with 95% confidence interval (CI). Time-to-event curves were estimated using the Kaplan–Meier method. The statistical analyses were performed two-tailed, and *p*-value < 0.05 was considered as the threshold for statistical significance. All statistical test were executed using R software version 3.6.3 (R software, R Foundation for Statistical Computing, Vienna, Austria).

## 3. Results

### 3.1. Baseline Characteristics

A total of 2025 TAVI patients enrolled in the REVASC-TAVI registry, with available data about the completeness of myocardial revascularization and clinical status at follow-up, were included in the present analysis. The median age was 82.4 years and 41.3% of patients were female. Predicted mortality risk according to the Society of Thoracic Surgeons (STS) score was 5.0%. Only 28.1% of patients had angina and 63.1% were in NHYA class III or IV. Baseline characteristics of patients according to PCI treatment or not, and according to vital status at 2 years, are reported in Table 1 and Supplementary Table S1, respectively.

### 3.2. TAVI Procedure Details

Most TAVI procedures were performed using the transfemoral access (95.1%) under local anesthesia (86.0%). The SAPIEN (Edwards LifeSciences, Irvine, CA, USA) (39.2%) transcatheter aortic valve (TAV) was the most used device. The procedural characteristics of patients according to CAD treatment, and according to vital status at 2 years, are reported in Supplementary Tables S2 and S3, respectively.

### 3.3. Coronary Artery Disease Details

Most patients had single-vessel CAD (56.1%). The vessel more commonly involved was left anterior descending (LAD) (64.3%), followed by right coronary artery (RCA) (46.3%). In 60.3% of patients, CAD involved proximal coronary segments, with 12.0% involving LM. Calcified CAD and coronary bifurcation involvement were observed in 22.6% and 26.3% of patients, respectively (Central illustration). The median BCIS-JS was 4 (IQR 2–6). The coronary disease characteristics of patients according to CAD treatment, and according to vital status at 2 years, are reported in Table 2 and Supplementary Table S4.

### 3.4. Percutaneous Coronary Intervention Details

**Scheme 1:**
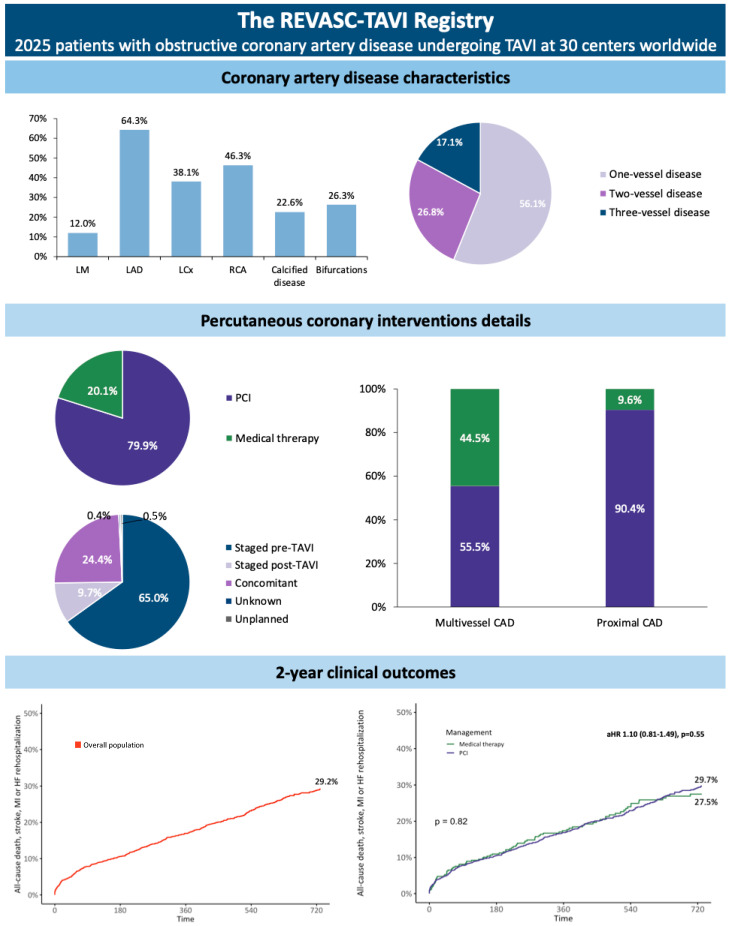
Coronary artery disease (CAD) and percutaneous coronary intervention (PCI) characteristics and outcomes of patients included in the REVASC-TAVI Registry. aHR, adjusted hazard ratio; LAD, left anterior descending; LM, left main; TAVI, transcatheter aortic valve implantation.

The characteristics of PCIs performed are reported in Table 3. Percutaneous coronary intervention was performed in 1617 patients (79.9%), with a total of 2014 lesions treated. Most patients underwent PCI in a separate session before TAVI (65.0%, n = 1052), whereas PCI was performed in the same session of TAVI or staged after the index procedure in 24.4% (n = 394) and 9.7% (n = 157) of cases, respectively (Scheme 1). The PCI procedures had high procedural success (97.4%) and were performed through a trans-femoral approach in 44.6% of cases.

Completeness of revascularization was achieved in 81.0% of cases undergoing PCI. The requirement of hemodynamic support was infrequent (1.1%). Similarly, the use of invasive imaging [intravascular ultrasound (IVUS) or optical coherence tomography (OCT) (7.0%)] or physiological assessment [fractional flow reserve (FFR) or instantaneous wave-free ratio (iFR) (9.5%)] was uncommon.

In 15.7% of cases, PCI was performed in coronary segments with stenosis greater than 90% of the vessel reference. Chronic total occlusion (CTO) PCIs were performed in 2.5% of cases. The coronary segments most frequently treated were mid (27.8%) and proximal LAD (23.1%). Coronary disease in more than one vessel and in proximal coronary segment was treated in 55.5% and 90.4%, respectively (Scheme 1).

### 3.5. Clinical Outcomes

**Figure 1:**
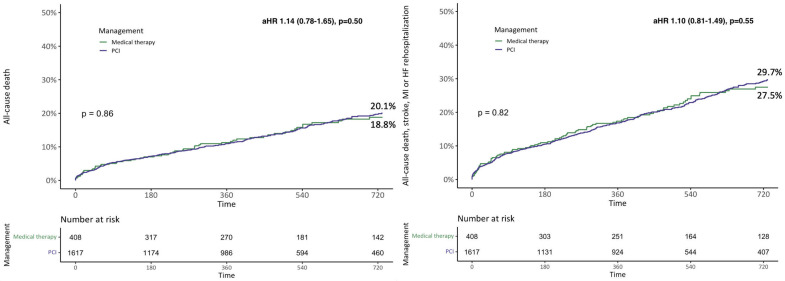
Two-year Kaplan–Meier survival curves for all-cause death and the composite of all-cause death, stroke, myocardial infarction (MI), and rehospitalization for heart failure (HF) according to the treatment received. aHR, adjusted hazard ratio; PCI, percutaneous coronary intervention.

**Figure 2:**
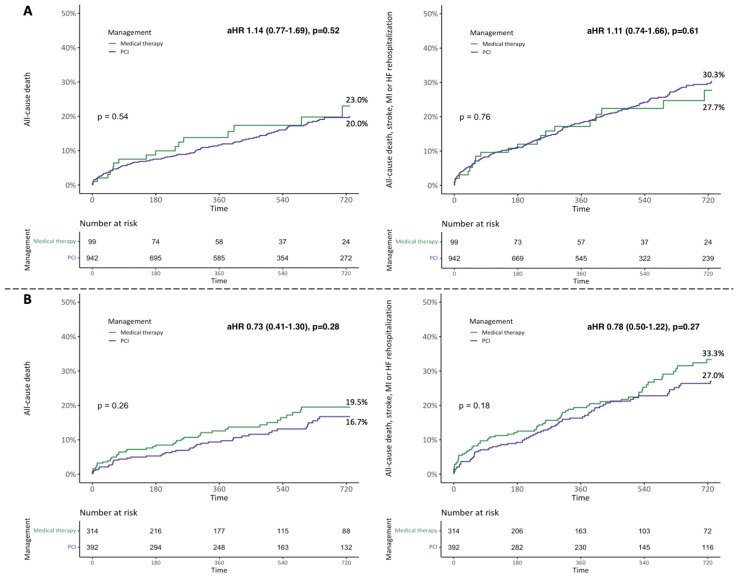
Two-year Kaplan–Meier survival curves for all-cause death and the composite of all-cause death, stroke, myocardial infarction (MI), and rehospitalization for heart failure (HF) according to the treatment received in different subgroups. (**A**) Proximal segment coronary artery disease (CAD). (**B**) Multivessel CAD. aHR, adjusted hazard ratio; PCI, percutaneous coronary intervention.

The outcomes of patients during hospitalization according to vital status at follow-up are reported in Supplementary Table S5. Considering the overall population, the Kaplan–Meier (KM) estimates for the 2-year all-cause death and the composite of all-cause death, stroke, MI, and rehospitalization for heart failure (HF) were 19.8% and 29.2%, respectively. All-cause death [20.1% vs. 18.8%, plog-rank = 0.86; adjusted hazard ratio (HR) 1.14 (0.78–1.65), *p* = 0.50] and the composite of all-cause death, stroke, MI, and HF rehospitalization [29.7% vs. 27.5%, plog-rank = 0.82; HR 1.10 (0.81–1.40), *p* = 0.55] were similar between patients receiving and those not receiving PCI at 2 years (Figure 1 and Central illustration). These results were consistent considering sub-groups of patients with CAD involving proximal coronary segments and more than one vessel (Figure 2).

Both all-cause death and the composite of all-cause death, stroke, MI, and HF rehospitalization did not differ according to the number of coronary vessels involved (plog-rank = 0.76 and plog-rank = 0.09, respectively) (Supplementary Figure S1).

No difference in all-cause death and in the composite of all-cause death, stroke, MI, and HF rehospitalization was found between patients receiving and those not receiving PCI in the case of one- (plog-rank = 0.36 and plog-rank = 0.22, respectively) or two-vessel CAD (plog-rank = 0.80 and plog-rank = 0.51, respectively). Despite the similar rate of all-cause death (plog-rank = 0.37), a lower rate of the composite of all-cause death, stroke, MI, and HF rehospitalization was observed in patients undergoing PCI in the case of three-vessel disease (32.5% vs. 44.5%, plog-rank =0.02) (Supplementary Figure S2).

Multivariable logistic regression analyses of baseline factors and of CAD characteristics with 2-year all-cause death or the composite of 2-year all-cause death, stroke, myocardial infarction, and HF rehospitalization were performed separately and are reported in Supplementary Tables S6–S9.

### 3.6. Predictors and Outcomes of Percutaneous Coronary Intervention

Patients treated with PCI more frequently had lesions involving LM (13.7% vs. 5.1%, *p* < 0.01), proximal coronary segments (64.5% vs. 54.7%, *p* < 0.01), or coronary bifurcations (30.4% vs. 12.0%, *p* < 0.01) (Table 2).

Evolut (Medtronic Inc, Minneapolis, MN, USA) was the most used TAVI platform in patients treated with medical therapy (43.7%), whereas SAPIEN 3 (Edwards Lifesciences, Irvine, CA, USA) was the one most used in patients receiving PCI (39.8%) (Supplementary Table S3).

Multivariable logistic regression analyses of baseline factors and CAD peculiarities with PCI were performed separately and are reported in Table 4 and Table 5.

Considering baseline characteristics, prior CABG [OR 0.40 (0.28–0.59), *p* < 0.01] and reduced left ventricular ejection fraction [OR 0.76 (0.55–1.05), *p* = 0.09] were found to be associated with a decreased probability of undergoing PCI, whereas prior PCI [OR 2.53 (1.95–3.30), *p* < 0.01] and angina (Canadian Cardiovascular Society class > 1) [OR 1.86 (1.39–2.50), *p* < 0.01] were independently associated with an increased probability of PCI.

Considering CAD peculiarities, the presence of lesions involving LM [OR 2.95 (1.73–5.31), *p* < 0.01], mid LAD [OR 1.55 (1.17–2.07), *p* < 0.01], proximal RCA [OR 1.55 (1.14–2.12), *p* = 0.01] or coronary bifurcation [OR 3.41 (2.36–5.02), *p* < 0.01] was independently associated with an increased probability of undergoing PCI. Contrarily, the presence of lesions in proximal left circumflex (LCx) [OR 0.61 (0.43–0.87), *p* = 0.01], diagonals [OR 0.45 (0.32–0.64), *p* < 0.01], or distal RCA/postero-lateral (PL)/posterior descending artery (PDA) [OR 0.66 (0.46–0.95), *p* = 0.03] was associated with a decreased probability of PCI.

## 4. Discussion

Clinical outcomes of patients undergoing TAVI with concomitant myocardium at risk due to coronary artery stenosis have been previously investigated in small studies [7,8,9,10,11].

The aim of this analysis from the international, multicenter REVASC-TAVI registry was to provide an updated overview of characteristics and outcomes of TAVI patients with obstructive CAD in the current clinical practice, delving into CAD peculiarities and the management of this population. The main findings were as follows:

(1) Most of patients had one-vessel CAD, with proximal coronary segments involved in about 60% of cases.
(2) About 80% of patients underwent PCI, most frequently performed before the TAVI procedure, with disease in proximal coronary segments being treated in about 90% of cases.
(3) Angina at baseline, lesions involving main tracts of coronary arteries, or coronary bifurcations led operators to perform PCI while patients with an involvement of distal or secondary vessels (i.e., diagonal or posterolateral branches) had a lower probability of receiving PCI.
(4) Physiologic assessment of coronary lesions was quite uncommon.
(5) The balloon-expandable SAPIEN 3 was the most used TAVI platform in patients treated with PCI.
(6) Patients receiving PCI had similar mid-term clinical outcomes compared to those treated conservatively.

This analysis represents the larger cohort study reporting clinical outcomes, with a granular assessment of CAD and its management, in patients undergoing TAVI with obstructive CAD in the real-world setting.

The population enrolled in the registry had a median age of 82 years and an intermediate mortality risk profile. Most patients had one-vessel CAD (56.1%). Proximal coronary segments were involved in 62.5% of patients, with 12.0% having an involvement of LM. Coronary disease was severely calcified or involved coronary bifurcations in 22.6% and 26.3%, respectively. About 80% of TAVI patients enrolled in the REVASC-TAVI registry underwent PCI, with a high success rate (97.4%).

The benefit of performing PCI in TAVI candidates is still debated. Current guidelines suggest considering PCI in case of a stenosis ≥70% in proximal coronary segments [12]. This recommendation has been translated from the surgical experience, where the invasiveness of therapy advocates the concomitant treatment of both AS and CAD, but poor evidence is available when dealing with patients undergoing TAVI [7,8,9,10,11].

Since latest guidelines, results from the first, ad hoc RCT have been published. The PercutAneous Coronary Implantation prIor to transcatheter aortic Valve Implantation (ACTIVATION) randomized clinical trial reported similar rates of death and rehospitalization at 1 year between TAVI patients with CAD treated and those not treated with PCI [4]. However, the results from the trial cannot be generalized to the current practice, as patients with angina or those with LM involvement were excluded.

A recent consensus of the European Association of Percutaneous Cardiovascular Interventions (EAPCI) society suggested that PCI should be performed only in those TAVI recipients with an involvement of proximal coronary segments, especially when presenting with acute coronary symptoms, angina, or sub-occlusive lesions [13].

Coronary interventions in the REVASC-TAVI registry were predominantly performed in a separate session before TAVI (65.0%). In patients with CAD affecting proximal coronary segments, PCI was performed in 90.4% of cases. Moreover, multivessel CAD was treated in 55.5% of cases.

These data suggest a certain degree of patient selection by operators when deciding to perform PCI, even if CAD had a large quote of myocardium at risk. Operators used to tailor the management of CAD and AS mainly according to clinical presentation, patients’ characteristics, and their lifespan. In fact, it should be considered that complex CAD may anticipate a challenging PCI procedure, with longer procedural times, a larger amount of contrast dye, and a higher risk of complications. On top of the potential benefits of PCI, the additional bleeding risk of anti-thrombotic therapy in this population should be also assessed.

In multivariable regression analyses investigating factors associated with PCI, we found that patients with angina at baseline or with CAD involving main coronary segments such as proximal RCA, mid LAD, or LM had a higher probability of receiving PCI. Contrarily, those patients with an involvement of distal or secondary vessels (i.e., diagonal or postero-lateral branches) had a lower probability of receiving PCI.

Interestingly, patients with lesions involving a proximal tract of circumflex artery had a lower probability of receiving PCI. This might be related to the unfavorable characteristics of the circumflex artery anatomy, as well as procedural implications and outcomes of left circumflex ostium PCI.

Moreover, the involvement of coronary bifurcations was associated with a higher probability of receiving PCI. Although the PCI of these lesions is expected to be more challenging, the treatment of coronary bifurcation might have been supported by the higher quote of myocardium at risk compared to single-vessel lesions.

The benefit of physiology-guided PCI is well established in patients affected by stable CAD, permitting to identify those lesions causing myocardial ischemia, and therefore to restrict interventions. However, in patients affected by severe AS undergoing TAVI, the role of an iFR/FFR assessment of CAD is still debated and needs to be further clarified [13]. As expected, the adoption of a physiological assessment of CAD in our registry was low (9.5% of coronary lesions). Ongoing, ad hoc validation studies and clinical trials (NOTION-3 and FAI-TAVI) are awaited to confirm the role of physiology-guided PCI in the TAVI population.

The use of balloon-expandable SAPIEN 3 was the most frequent TAVI device in patients undergoing PCI, whereas the self-expanding Evolut was the most used platform in patients treated conservatively. We might speculate that the SAPIEN 3 valve was preferred in patients undergoing PCI because this device has a design that guarantees easier coronary re-access. Nevertheless, it should be considered that patients treated conservatively may necessitate PCI after TAVI due to CAD progression or acute coronary syndromes. Coronary re-access after TAVI might be difficult in some patients treated with supra-annular, small-cell-design TAVI devices and unfavorable aortic root characteristics, and challenges in obtaining coronary ostium engagement may potentially make PCI more challenging, or even impossible in these patients [14,15,16].

At 2 years, the clinical outcomes of patients enrolled in the registry were in line with those reported by other TAVI series of elderly patients at intermediate risk [17]. The rates of 2-year all-cause death and the composite of all-cause death, stroke, MI, and HF rehospitalization did not differ between patients receiving and those not receiving PCI, regardless of the involvement of proximal coronary segments or more than one coronary artery. Nevertheless, revascularization might play a role in patients with three-vessel CAD undergoing TAVI. Indeed, despite similar 2-year all-cause death, these patients had a lower rate of the composite of all-cause death, stroke, MI, and HF rehospitalization when receiving PCI.

We cannot conclude that the benefit of revascularization may become significant in the long term, and this should be considered especially when dealing with younger patients with a long life-expectancy.

## 5. Conclusions

Obstructive CAD in patients undergoing TAVI presented more commonly as a single-vessel disease with the involvement of proximal coronary tracts. It was more commonly treated with PCI, performed in more than half of patients before the TAVI procedure. Moreover, patients receiving a revascularization strategy had similar mid-term clinical outcomes to those receiving medical treatment. The SAPIEN platform was the most used TAVI device in patients treated with PCI. Patients with angina or CAD in main coronary segments were more likely to receive PCI, whereas patients with CAD involving proximal left circumflex artery were more likely to be treated conservatively. Ad hoc, prospective studies with longer term follow-up are required to confirm the findings of this study.

## Figures and Tables

**Table 1:** Baseline characteristics of patients undergoing and those not undergoing PCI.

	Overall (n = 2025)	No PCI (n = 408)	PCI (n = 1617)	*p*-Value
Sex, male n (%)	1188 (58.7)	250 (61.3)	938 (58.0)	0.40
Age, median [IQR]	82.4 [78.0, 85.8]	83.0 [79.0, 86.0]	82.2 [78.0, 85.3]	0.12
Body mass index, median [IQR]	26.5 [23.8, 29.4]	26.7 [23.9, 29.8]	26.5 [23.7, 29.1]	0.29
Hypertension, n (%)	1719 (84.9)	341 (83.6)	1378 (85.2)	0.51
Diabetes, n (%)	648 (32.0)	132 (32.4)	516 (31.9)	0.26
Peripheric artery disease, n (%)	338 (16.7)	68 (16.7)	270 (16.7)	0.12
COPD, n (%)	319 (15.8)	67 (16.4)	252 (15.6)	0.06
eGFR, ml/min median [IQR]	55.1 [43.0, 64.5]	53.5 [39.6, 67.1]	55.1 [44.0, 64.0]	0.04
Prior CABG, n (%)	195 (9.6)	61 (15.0)	134 (8.3)	<0.01
Prior PCI, n (%)	963 (47.6)	107 (26.2)	856 (52.9)	<0.01
Prior myocardial infarction, n (%)	405 (20.0)	93 (22.8)	312 (19.3)	0.04
Prior stroke, n (%)	169 (8.3)	22 (5.4)	147 (9.1)	<0.01
Prior pacemaker, n (%)	178 (8.8)	45 (11.0)	133 (8.2)	0.01
Prior SAVR, n (%)	39 (1.9)	7 (1.7)	32 (2.0)	<0.01
Bicuspid aortic valve, n (%)	80 (4.0)	11 (2.7)	69 (4.3)	<0.01
CCS class > 1, n (%)	444 (28.1)	73 (19.2)	371 (30.9)	<0.01
NYHA class > 2, n (%)	1274 (63.1)	262 (64.2)	1012 (62.8)	0.61
Atrial fibrillation, n (%)	541 (26.7)	102 (25.0)	439 (27.1)	0.04
STS mortality score, % median [IQR]	5.0 [3.1, 5.1]	4.3 [2.8, 5.4]	5.0 [3.2, 5.1]	0.01
LVEF, % median [IQR]	55.0 [45.0, 61.0]	55.0 [43.8, 60.0]	56.0 [47.0, 62.0]	<0.01
LVEF < 40%, n (%)	300 (14.8)	73 (17.9)	227 (14.0)	0.05
Aortic mean gradient, mmHg, median [IQR]	44.0 [36.0, 51.0]	44.4 [38.0, 51.0]	44.0 [36.0, 51.0]	0.22
Aortic valve area, cm^2^ median [IQR]	0.7 [0.6, 0.8]	0.7 [0.6, 0.8]	0.7 [0.6, 0.8]	0.23
Dual antiplatelet therapy, n (%)	745 (36.8)	23 (5.6)	722 (44.7)	<0.01
Dual anti-thrombotic therapy, n (%)	145 (7.2)	23 (5.6)	122 (7.5)	<0.01
Triple anti-thrombotic therapy, n (%)	107 (5.3)	0 (0.0)	107 (6.6)	<0.01

CABG, coronary artery bypass graft; CCS, Canadian Cardiovascular Society; COPD, Chronic obstructive pulmonary disease; eGFR, estimated glomerular filtration rate; IQR, interquartile range; LVEF, Left ventricular ejection fraction; NYHA, New York Heart Association; PCI, Percutaneous coronary intervention; SAVR, Surgical aortic valve replacement; STS, Society of Thoracic Surgeons.

**Table 2:** Characteristics of coronary artery disease (CAD) in patients receiving and those not receiving percutaneous coronary intervention (PCI).

	Overall (n = 2025)	No PCI (n = 408)	PCI (n = 1617)	*p*-Value
Diseased vessels, n (%)				0.81
One	1137 (56.1)	227 (55.6)	910 (56.3)	
Two	542 (26.8)	107 (26.2)	435 (26.9)	
Three or more	346 (17.1)	74 (18.1)	272 (16.8)	
Right dominance, n (%)	1634 (83.7)	328 (81.8)	1306 (84.2)	0.26
Multivessel CAD, n (%)	403 (22.6)	181 (44.4)	707 (43.7)	0.82
Proximal CAD, n (%)	432 (26.3)	223 (54.7)	1043 (64.5)	<0.01
Calcific disease, n (%)	888 (43.9)	73 (19.8)	330 (23.3)	0.18
Bifurcation involved, n (%)	1266 (62.5)	44 (12.0)	388 (30.4)	<0.01
Syntax score, median [IQR]	8 [5, 13]	7 [4, 11]	8 [5, 14]	<0.01
BCIS jeopardy score, median [IQR]	4 [2, 6]	4 [2, 6]	4 [2, 8]	<0.01
Residual BCIS jeopardy score, median [IQR]	0 [0, 2]	4 [2, 6]	0 [0, 0]	<0.01
**Coronary segments involved**				
LM, n (%)	242 (12.0)	21 (5.1)	221 (13.7)	<0.01
LAD, n (%)	1302 (64.3)	265 (65.0)	1037 (64.1)	0.77
Proximal LAD, n (%)	615 (30.4)	103 (25.2)	512 (31.7)	0.01
Mid LAD, n (%)	768 (37.9)	133 (32.6)	635 (39.3)	0.01
Distal LAD, n (%)	146 (7.2)	30 (7.4)	116 (7.2)	0.92
Diagonal, n (%)	298 (14.7)	87 (21.3)	211 (13.0)	<0.01
LCx, n (%)	771 (38.1)	184 (45.1)	587 (36.3)	0.00
Proximal LCx, n (%)	357 (17.6)	84 (20.6)	273 (16.9)	0.08
Mid LCx, n (%)	232 (11.5)	45 (11.0)	187 (11.6)	0.80
Distal LCx/PDA, n (%)	96 (4.7)	25 (6.1)	71 (4.4)	0.15
Obtuse marginal, n (%)	288 (14.2)	78 (19.1)	210 (13.0)	0.00
RCA, n (%)	937 (46.3)	188 (46.1)	749 (46.3)	0.96
Proximal RCA, n (%)	551 (27.2)	90 (22.1)	461 (28.5)	0.01
Mid RCA, n (%)	404 (20.0)	82 (20.1)	322 (19.9)	0.95
Distal RCA/PL/PDA, n (%)	254 (12.5)	71 (17.4)	183 (11.3)	0.00
Venous/arterial graft, n (%)	76 (3.8)	17 (4.2)	59 (3.6)	0.66

LAD, Left anterior descending; LCx, Left circumflex; LM, Left main; PDA, Posterior descending artery; PL, Postero-lateral; RCA, Right coronary artery.

**Table 3:** Details of percutaneous coronary interventions performed in the REVASC-TAVI registry.

	Overall Lesions (n = 2014)
Target vessel stenosis, n (%)	
NA	9 (0.5)
> 70%	1587 (81.3)
> 90%	306 (15.7)
CTO	49 (2.5)
PCI access (%)	
Right radial	938 (48.8)
Left radial	75 (3.9)
Femoral	910 (47.3)
NA	1 (0.1)
iFR/FFR use, n (%)	159 (9.5)
IVUS/OCT use, n (%)	117 (7.0)
Hemodynamic support, n (%)	22 (1.1)
Atherectomy, n (%)	87 (4.5)
LM, n (%)	229 (11.4)
LAD, n (%)	912 (45.3)
Proximal LAD, n (%)	465 (23.1)
Mid LAD, n (%)	560 (27.8)
Distal LAD, n (%)	77 (3.8)
Diagonal, n (%)	107 (5.3)
LCx, n (%)	342 (17.0)
Proximal LCx, n (%)	198 (9.8)
Mid LCx, n (%)	133 (6.6)
Distal LCx, n (%)	48 (2.4)
Obtuse marginal, n (%)	127 (6.3)
RCA, n (%)	585 (29.0)
Proximal RCA, n (%)	358 (17.8)
Mid RCA, n (%)	246 (12.2)
Distal RCA/PL/PDA, n (%)	123 (6.1)
Venous arterial graft, n (%)	41 (2.0)
Procedural success, n (%)	1924 (97.4)
Crossing difficulty, n (%)	49 (2.6)

CTO, Chronic total occlusion; FFR, Fractional flow reserve; iFR, Instantaneous wave-free ratio; IVUS, Intravascular ultrasound; LAD, Left anterior descending; LCx, Left circumflex; LM, Left main; NA, Not available; OCT, Optical coherence tomography; PCI, Percutaneous coronary intervention; PDA, Posterior descending artery; PL, Postero-lateral; RCA, Right coronary artery.

**Table 4:** Multivariable regression analyses of baseline characteristics associated with percutaneous coronary intervention.

Baseline Characteristics	OR (95% CI)	*p*-Value
Female	1.17 (0.91–1.50)	0.22
Age, year	0.99 (0.97–1.01)	0.34
Diabetes	0.86 (0.66–1.11)	0.24
eGFR, mL/min	1.00 (1.00–1.01)	0.43
Prior CABG	0.40 (0.28–0.59)	<0.01
Prior PCI	2.53 (1.95–3.30)	<0.01
CCS > 1	1.86 (1.39–2.50)	<0.01
NYHA > 2	0.96 (0.74–1.24)	0.75
Atrial fibrillation	1.06 (0.80–1.40)	0.70
LVEF < 40%	0.76 (0.55–1.05)	0.09

CABG, coronary artery bypass graft; CCS, Canadian Cardiovascular Society; CI, confidence interval; eGFR, estimated glomerular filtration rate; LVEF, left ventricular ejection fraction; NYHA, New York Heart Association; OR, odds ratio; PCI, percutaneous coronary intervention.

**Table 5:** Multivariable regression analyses of coronary artery disease (CAD) characteristics associated with percutaneous coronary intervention.

CAD Characteristics	OR (95% CI)	*p*-Value
LM	2.95 (1.73–5.31)	<0.01
Proximal LAD	1.30 (0.97–1.75)	0.08
Mid LAD	1.55 (1.17–2.07)	<0.01
Distal LAD	1.08 (0.69–1.76)	0.74
Diagonal	0.45 (0.32–0.64)	<0.01
Proximal LCx	0.61 (0.43–0.87)	0.01
Mid LCx	1.27 (0.84–1.97)	0.31
Distal LCx/PDA	0.75 (0.43–1.32)	0.30
Obtuse marginal	0.73 (0.51–1.05)	0.09
Proximal RCA	1.55 (1.14–2.12)	0.01
Mid RCA	1.30 (0.94–1.81)	0.11
Distal RCA/PL/PDA	0.66 (0.46–0.95)	0.03
Calcific disease	1.12 (0.82–1.54)	0.47
Bifurcation	3.41 (2.36–5.02)	<0.01
Multivessel CAD	0.90 (0.63–1.30)	0.59

CI, confidence interval; LM, Left main; LAD, Left anterior descending; LCx, Left circumflex; RCA, Right coronary artery; OR, odds ratio; PL, Postero-lateral; PDA, Posterior descending artery.

## Data Availability

The data presented in this study are available on request from the corresponding author. The data are not publicly available due to privacy.

## Supplementary Materials

The following supporting information can be downloaded at: https://www.mdpi.com/article/10.3390/jcm13123497/s1, Figure S1. Two-year Kaplan-Meier survival curves for all-cause death and the composite of all-cause death, stroke, myocardial infarction (MI) or rehospitalization for heart failure (HF) according to the number of vessels involved. Figure S2. Two-year Kaplan-Meier survival curves for all-cause death and the composite of all-cause death, stroke, myocardial infarction (MI) or rehospitalization for heart failure (HF) according to the treatment received in different subgroups. (A) One-vessel coronary artery disease (CAD); (B) Two-vessel CAD; (C) Three-vessel CAD. PCI, percutaneous coronary intervention. Table S1. Baseline characteristics of patients according to vital status at 2 years. AF, Atrial fibrillation; AVA, Aortic valve area; BMI, Body mass index; CABG, coronary artery bypass graft; CCS, Canadian Cardiovascular Society; COPD, Chronic obstructive pulmonary disease; DAT, dual anti-thrombotic therapy; DAPT, dual antiplatelet therapy; eGFR, estimated glomerular filtration rate; IQR, interquartile range; LVEF, Left ventricular ejection fraction; MI, Myocardial infarction; NA, Not available; NYHA, New York Heart Association; PAD, Peripheric artery disease; PCI, Percutaneous coronary intervention; SAVR, Surgical aortic valve replacement; sPAP, systolic pulmonary artery pressure; STS, Society of Thoracic Surgeon; TAT, triple anti-thrombotic therapy. Table S2. Procedural characteristics of patients according to vital status at 2 years. NA, not available; SAT, supra-aortic trunks; TAV, transcatheter aortic valve. Table S3. Procedural characteristics of patients undergoing or not percutaneous coronary intervention (PCI). NA, not available; SAT, supra-aortic trunks; TAV, transcatheter aortic valve. Table S4. Characteristics of coronary artery disease according to patients’ vital status at 2 years. LAD, Left Anterior Descendent; LCx, Left circumflex; LM, Left Main; PDA, Posterior descending artery; PL, postero-lateral; RCA, Right coronary artery. Table S5. In-hospital outcomes of patients according to vital status at 2 years. AF, atrial fibrillation; AKI, acute kidney injury; LBBB, left bungle branch block; MI, myocardial infarction; PPI, permanent pacemaker implantation; PVR, para-valvular regurgitation. Table S6. Multivariable regression analyses of baseline characteristics associated with 2-year all-cause death. AF, atrial fibrillation; CABG, coronary artery bypass graft; CCS, canadian cardiovascular society; CI, confidence interval; COPD, chronic obstructive pulmonary disease; eGFR, estimated glomerular filtration rate; LVEF, left ventricular ejection fraction; MI, myocardial infarction; NA, not available; NYHA, New York Heart Association; OR, odds ratio; PAD, peripheric artery disease; PCI, percutaneous coronary intervention. Table S7. Multivariable regression analyses of coronary artery disease (CAD) characteristics associated with 2-year all-cause death. CI, confidence interval; LM, Left Main; LAD, Left Anterior Descendent; LCx, Left circumflex; RCA, Right coronary artery; OR, odds ratio; PL, postero-lateral; PDA, Posterior descending artery. Table S8. Multivariable regression analyses of baseline characteristics associated with the composite of 2-year all-cause death, stroke, myocardial infarction and rehospitalization for heart failure. AF, atrial fibrillation; BMI, body mass index; CABG, coronary artery bypass graft; CCS, canadian cardiovascular society; CI, confidence interval; COPD, chronic obstructive pulmonary disease; eGFR, estimated glomerular filtration rate; LVEF, left ventricular ejection fraction; MI, myocardial infarction; NA, not available; NYHA, New York Heart Association; OR, odds ratio; PAD, peripheric artery disease; PCI, percutaneous coronary intervention. Table S9. Multivariable regression analyses of CAD characteristics associated with the composite of 2-year all-cause death, stroke, myocardial infarction and rehospitalization for heart failure. CI, confidence interval; LM, Left Main; LAD, Left Anterior Descendent; LCx, Left circumflex; RCA, Right coronary artery; OR, odds ratio; PL, postero-lateral; PDA, Posterior descending artery.
