# Supplementary material for: Characterization and Management of Stable Coronary Artery Disease in Patients Undergoing Transcatheter Aortic Valve Implantation
Source: J Clin Med. 2024 Jun 14;13(12):3497. doi: 10.3390/jcm13123497 (PMC11204567; doi:10.3390/jcm13123497)
Supplement: Supplementary file 1 [file jcm-13-03497-s001.zip › jcm-2975690-supplementary.pdf]

Supplementary figure 1. Two-year Kaplan-Meier survival curves for all-cause death and the composite of all-cause death, stroke, myocardial infarction (MI) or rehospitalization for heart failure (HF) according to the number of vessels involved.

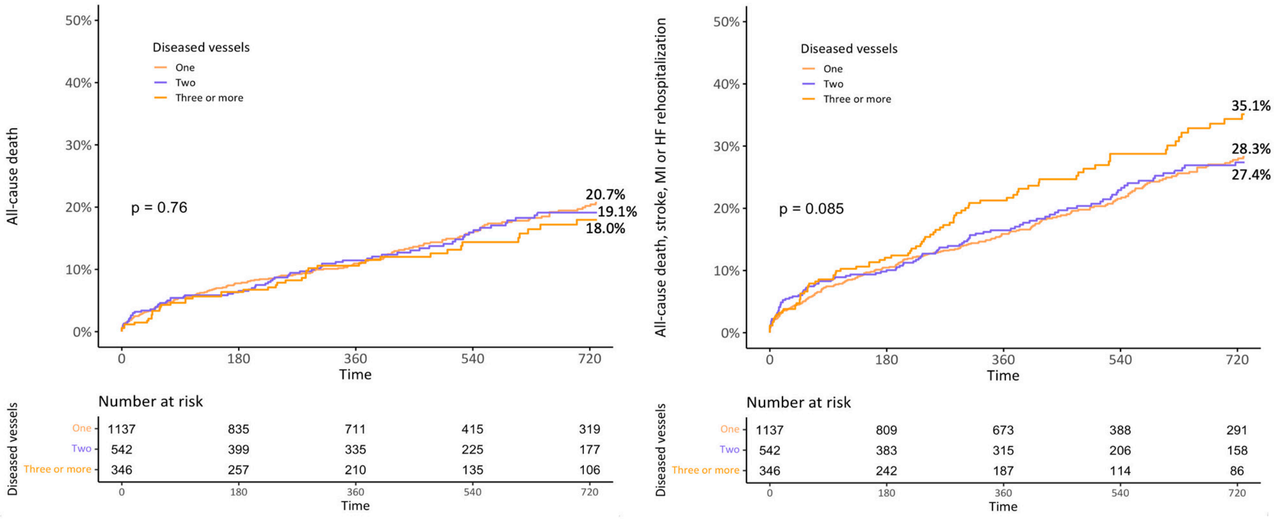

Supplementary figure 2. Two-year Kaplan-Meier survival curves for all-cause death and the composite of all-cause death, stroke, myocardial infarction (MI) or rehospitalization for heart failure (HF) according to the treatment received in different subgroups. A) One-vessel coronary artery disease (CAD); B) Two-vessel CAD; C) Three-vessel CAD. *PCI, percutaneous coronary intervention*

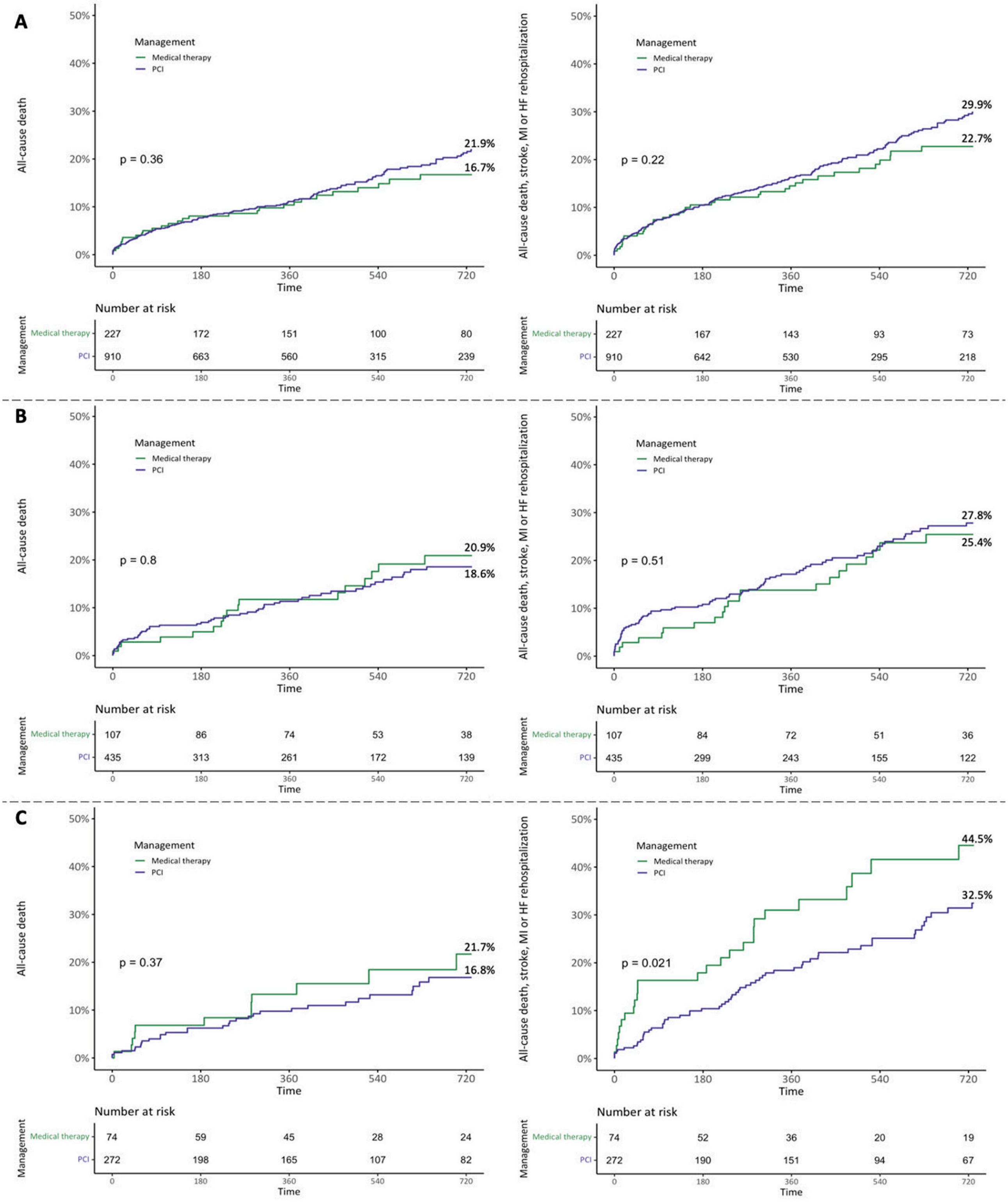

Supplementary table 1. Baseline characteristics of patients according to vital status at 2 years. *AF, Atrial fibrillation; AVA, Aortic valve area; BMI, Body mass index; CABG, coronary artery bypass graft; CCS, Canadian Cardiovascular Society; COPD, Chronic obstructive pulmonary disease; DAT, dual anti-thrombotic therapy; DAPT, dual antiplatelet therapy; eGFR, estimated glomerular filtration rate; IQR, interquartile range; LVEF, Left ventricular ejection fraction; MI, Myocardial infarction; NA, Not available; NYHA, New York Heart Association; PAD, Peripheric artery disease; PCI, Percutaneous coronary intervention; SAVR, Surgical aortic valve replacement; sPAP, systolic pulmonary artery pressure; STS, Society of Thoracic Surgeon; TAT, triple anti-thrombotic therapy.*

	**Alive (n=1751)**	**Dead (n=274)**	**p-val**
Sex, male n (%)	1026 (58.6)	162 (59.1)	0.59
Age, median [IQR]	82.0 [78.0, 85.3]	83.0 [79.0, 86.0]	0.02
BMI, median [IQR]	26.6 [23.9, 29.4]	25.9 [23.2, 28.7]	0.03
Hypertension, n (%)	1492 (85.2)	227 (82.8)	0.24
Diabetes, n (%)	549 (31.4)	99 (36.1)	0.24
PAD, n (%)	286 (16.3)	52 (19.0)	0.21
COPD, n (%)	259 (14.8)	60 (21.9)	<0.01
eGFR, ml/min median [IQR]	55.1 [44.0, 65.7]	51.1 [34.7, 59.0]	<0.01
Prior CABG, n (%)	169 (9.7)	26 (9.5)	0.39
Prior PCI, n (%)	826 (47.2)	137 (50.0)	0.47
Prior MI, n (%)	341 (19.5)	64 (23.4)	0.33
Prior stroke, n (%)	138 (7.9)	31 (11.3)	<0.01
Prior pacemaker, n (%)	155 (8.9)	23 (8.4)	0.57
Prior SAVR, n (%)	37 (2.1)	2 (0.7)	0.08
Bicuspid aortic valve, n (%)	67 (3.8)	13 (4.7)	0.24
CCS class >1, n (%)	392 (28.2)	52 (27.2)	0.86
NYHA class >2, n (%)	1081 (61.9)	193 (70.7)	<0.01
AF, n (%)	440 (25.1)	101 (36.9)	<0.01
STS Mortality score, % median [IQR]	5.0 [3.1, 5.0]	5.0 [3.6, 6.9]	<0.01
LVEF, % median [IQR]	55.0 [47.0, 61.0]	55.0 [45.0, 60.0]	0.23
Aortic mean gradient, mmHg, median [IQR]	44.0 [37.0, 51.0]	42.0 [31.0, 48.0]	<0.01
AVA, cm^2 median [IQR]	0.7 [0.6, 0.8]	0.7 [0.6, 0.8]	0.94
DAPT, n (%)	650 (37.1)	95 (34.7)	0.46
DAT, n (%)	128 (7.3)	17 (6.2)	0.45
TAT, n (%)	89 (5.1)	18 (6.6)	0.25

Supplementary table 2. Procedural characteristics of patients according to vital status at 2 years. *NA, not available; SAT, supra-aortic trunks; TAV, transcatheter aortic valve*

	**Overall (n=2025)**	**Alive (n=1751)**	**Dead (n=274)**	**p-val**
**Vascular access, n (%)**				0.05
Trans-femoral	1914 (95.1)	1660 (95.3)	254 (93.4)	
Trans-apical	36 (1.8)	32 (1.8)	4 (1.5)	
Trans-subclavian	46 (2.3)	34 (2.0)	12 (4.4)	
Direct aortic	14 (0.7)	13 (0.7)	1 (0.4)	
Others	2 (0.1)	2 (0.1)	0 (0.0)	
NA	1 (0.0)	0 (0.0)	1 (0.4)	
SAT protection, n (%)	91 (4.8)	87 (5.3)	4 (1.6)	0.01
**TAV type, n (%)**				0.20
SAPIEN 3/Ultra	770 (38.2)	677 (38.8)	93 (34.1)	
SAPIEN XT	21 (1.0)	16 (0.9)	5 (1.8)	
Evolut R/PRO/PRO+	676 (33.5)	578 (33.2)	98 (35.9)	
CoreValve	76 (3.8)	68 (3.9)	8 (2.9)	
Portico	127 (6.3)	106 (6.1)	21 (7.7)	
Lotus	32 (1.6)	26 (1.5)	6 (2.2)	
Acurate NEO/NEO 2	267 (13.2)	231 (13.3)	36 (13.2)	
Allegra	11 (0.5)	8 (0.5)	3 (1.1)	
Others	36 (1.8)	33 (1.9)	3 (1.1)	
Anesthesia, local n (%)	1730 (86.0)	1509 (86.4)	221 (83.1)	0.27
Post dilatation, n (%)	411 (22.2)	351 (22.0)	60 (23.7)	0.52
Need 2nd TAV, n (%)	34 (1.7)	28 (1.6)	6 (2.2)	0.45
Contrast dye, median [IQR]	132.0 [93.3, 210.0]	130.0 [90.3, 201.0]	160.0 [98.5, 240.0]	0.02

Supplementary table 3. Procedural characteristics of patients undergoing or not percutaneous coronary intervention (PCI). *NA, not available; SAT, supra-aortic trunks; TAV, transcatheter aortic valve*

	**No PCI (n=408)**	**PCI (n=1617)**	**p-val**
**Vascular access, n (%)**			0.10
Trans-femoral	397 (97.8)	1517 (94.4)	
Trans-apical	5 (1.2)	31 (1.9)	
Trans-subclavian	3 (0.7)	43 (2.7)	
Direct aortic	1 (0.2)	13 (0.8)	
Others	0 (0.0)	2 (0.1)	
NA	0 (0.0)	1 (0.1)	
SAT protection, n (%)	16 (4.1)	75 (5.0)	0.51
**TAV type, n (%)**			<0.01
SAPIEN 3/Ultra	130 (31.9)	640 (39.8)	
SAPIEN XT	9 (2.2)	12 (0.7)	
Evolut R/PRO/PRO+	178 (43.7)	498 (31.0)	
CoreValve	18 (4.4)	58 (3.6)	
Portico	15 (3.7)	112 (7.0)	
Lotus	14 (3.4)	18 (1.1)	
Acurate NEO/NEO 2	35 (8.6)	232 (14.4)	
Allegra	3 (0.7)	8 (0.5)	
Others	5 (1.2)	31 (1.9)	
Anesthesia, local n (%)	338 (84.1)	1392 (86.5)	<0.01
Post dilatation, n (%)	90 (22.2)	321 (22.2)	1.00
Need 2nd TAV, n (%)	12 (2.9)	22 (1.4)	0.05
Contrast dye, median [IQR]	151.0 [100.0, 220.0]	130.0 [90.0, 205.0]	<0.01

Supplementary table 4. Characteristics of coronary artery disease according to patients' vital status at 2 years. *LAD, Left Anterior Descendent; LCx, Left circumflex; LM, Left Main; PDA, Posterior descending artery; PL, postero-lateral; RCA, Right coronary artery*

	**Overall (n=2025)**	**Alive (n=1751)**	**Dead (n=274)**	**p-val**
Diseased vessels, n (%)				0.83
One	1137 (56.1)	980 (56.0)	157 (57.3)	
Two	542 (26.8)	468 (26.7)	74 (27.0)	
Three or more	346 (17.1)	303 (17.3)	43 (15.7)	
Right dominance, n (%)	1634 (83.7)	1412 (83.5)	222 (85.1)	0.59
Calcific disease, n (%)	403 (22.6)	353 (22.7)	50 (21.7)	0.800
Bifurcation involved, n (%)	432 (26.3)	388 (26.9)	44 (22.0)	0.15
Multivessel CAD, n (%)	888 (43.9)	771 (44.0)	117 (42.7)	0.70
Proximal CAD, n (%)	1266 (62.5)	1090 (62.3)	176 (64.2)	0.55
Syntax score, median [IQR]	8 [5,13]	8 [5,14]	7 [4,13]	0.13
BCIS jeopardy score, median [IQR]	4 [2, 6]	4 [2, 6]	5 [4, 6]	0.44
Residual BCIS jeopardy score, median [IQR]	0 [0, 2]	0 [0, 2]	0 [0, 4]	0.47
**Coronary segments involved**				
LM, n (%)	242 (12.0)	209 (11.9)	33 (12.0)	0.92
LAD, n (%)	1302 (64.3)	1137 (64.9)	165 (60.2)	0.14
Proximal LAD, n (%)	615 (30.4)	529 (30.2)	86 (31.4)	0.72
Mid LAD, n (%)	768 (37.9)	674 (38.5)	94 (34.3)	0.20
Distal LAD, n (%)	146 (7.2)	130 (7.4)	16 (5.8)	0.38
Diagonal, n (%)	298 (14.7)	260 (14.8)	38 (13.9)	0.72
LCx, n (%)	771 (38.1)	665 (38.0)	106 (38.7)	0.84
Proximal LCx, n (%)	357 (17.6)	305 (17.4)	52 (19.0)	0.55
Mid LCx, n (%)	232 (11.5)	203 (11.6)	29 (10.6)	0.68
Distal LCx/PDA, n (%)	96 (4.7)	80 (4.6)	16 (5.8)	0.36
Obtuse marginal, n (%)	288 (14.2)	248 (14.2)	40 (14.6)	0.85
RCA, n (%)	937 (46.3)	810 (46.3)	127 (46.4)	1.00
Proximal RCA, n (%)	551 (27.2)	487 (27.8)	64 (23.4)	0.13
Mid RCA, n (%)	404 (20.0)	351 (20.0)	53 (19.3)	0.87
Distal RCA/PL/PDA, n (%)	254 (12.5)	217 (12.4)	37 (13.5)	0.62
Venous/arterial graft, n (%)	76 (3.8)	67 (3.8)	9 (3.3)	0.86

Supplementary table 5. In-hospital outcomes of patients according to vital status at 2 years. *AF, atrial fibrillation; AKI, acute kidney injury; LBBB, left bungle branch block; MI, myocardial infarction; PPI, permanent pacemaker implantation; PVR, para-valvular regurgitation.*

	**Overall (n=2025)**	**Alive (n=1751)**	**Dead (n=274)**	**p-val**
Death, n (%)	47 (2.3)	0 (0.0)	43 (15.9)	<0.01
Cardiovascular death, n (%)	32 (1.6)	0 (0.0)	30 (11.5)	<0.01
Disabling Stroke, n (%)	25 (1.2)	18 (1.0)	7 (2.6)	0.07
Not disabling Stroke, n (%)	24 (1.4)	22 (1.4)	2 (0.8)	0.76
MI, n (%)	16 (0.8)	9 (0.5)	7 (2.7)	<0.01
PPI, n (%)	224 (12.4)	184 (11.8)	40 (16.3)	0.06
New onset LBBB, n (%)	264 (14.6)	230 (14.7)	34 (13.9)	0.77
New onset AF, n (%)	51 (2.9)	42 (2.8)	9 (3.8)	0.41
Minor Bleeding, n (%)	161 (8.0)	133 (7.7)	28 (10.4)	0.15
Major Bleeding, n (%)	103 (5.1)	85 (4.9)	18 (6.7)	0.24
Life-Threatening Bleeding, n (%)	40 (2.0)	26 (1.5)	14 (5.2)	<0.01
Major Vascular Complication, n (%)	97 (4.8)	77 (4.4)	20 (7.4)	0.05
Minor Vascular Complication, n (%)	163 (8.1)	142 (8.2)	21 (7.8)	0.91
AKI, n (%)				<0.01
Stage 1	132 (6.7)	106 (6.2)	26 (9.8)	
Stage 2	31 (1.6)	26 (1.5)	5 (1.9)	
Stage 3	32 (1.6)	19 (1.1)	13 (4.9)	
Aortic mean gradient, median [IQR]	8.0 [6.0, 11.0]	8.0 [6.0, 11.0]	8.0 [6.0, 11.0]	0.12
PVR grade, n (%)				0.22
None/trivial	865 (45.9)	760 (45.9)	105 (45.5)	
Mild	922 (48.9)	810 (48.9)	112 (48.5)	
Moderate/severe	99 (5.2)	85 (5.1)	14 (6.1)	
Length of stay, days, median [IQR]	5.0 [2.0, 7.0]	5.0 [2.0, 7.0]	5.0 [2.0, 8.0]	0.63

Supplementary table 6. Multivariable regression analyses of baseline characteristics associated with 2-year all-cause death. *AF, atrial fibrillation; CABG, coronary artery bypass graft; CCS, canadian cardiovascular society; CI, confidence interval; COPD, chronic obstructive pulmonary disease; eGFR, estimated glomerular filtration rate; LVEF, left ventricular ejection fraction; MI, myocardial infarction; NA, not available; NYHA, New York Heart Association; OR, odds ratio; PAD, peripheric artery disease; PCI, percutaneous coronary intervention.*

Baseline characteristics	OR (95% CI)	p-val
Female	1.04 (0.75-1.45)	0.81
Age, year	1.01 (0.99-1.04)	0.30
Diabetes	1.20 (0.86-1.66)	0.29
PAD	1.16 (0.77-1.72)	0.47
COPD	1.76 (1.20-2.56)	<0.01
eGFR, ml/min	0.98 (0.97-0.99)	<0.01
Prior CABG	1.06 (0.60-1.79)	0.83
Prior PCI	0.83 (0.59-1.17)	0.29
Prior MI	1.39 (0.92-2.06)	0.11
Prior stroke	1.40 (0.81-2.32)	0.20
Prior pacemaker	1.02 (0.58-1.69)	0.95
CCS > 1	0.93 (0.65-1.32)	0.68
NYHA > 2	1.87 (1.30-2.74)	<0.01
AF	1.65 (1.18-2.30)	<0.01
LVEF < 40 %	0.87 (0.56-1.32)	0.53

Supplementary table 7. Multivariable regression analyses of coronary artery disease (CAD) characteristics associated with 2-year all-cause death. *CI, confidence interval; LM, Left Main; LAD, Left Anterior Descendent; LCx, Left circumflex; RCA, Right coronary artery; OR, odds ratio; PL, postero-lateral; PDA, Posterior descending artery.*

CAD characteristics	OR (95% CI)	p-val
LM	0.95 (0.55-1.59)	0.85
Proximal LAD	1.27 (0.90-1.79)	0.16
Mid LAD	0.90 (0.64-1.26)	0.55
Distal LAD	0.81 (0.42-1.43)	0.49
Diagonal	1.01 (0.64-1.55)	0.97
Proximal LCx	1.24 (0.81-1.87)	0.32
Mid LCx	1.13 (0.68-1.81)	0.64
Distal LCx/PDA	1.31 (0.65-2.47)	0.42
Obtuse marginal	1.04 (0.65-1.62)	0.86
Proximal RCA	0.83 (0.56-1.20)	0.33
Mid RCA	0.91 (0.60-1.35)	0.65
Distal RCA/PL/PDA	1.39 (0.88-2.16)	0.15
Calcific disease	0.83 (0.56-1.21)	0.34
Bifurcation	0.70 (0.47-1.04)	0.09
Multivessel CAD	0.91 (0.59-1.41)	0.67

Supplementary table 8. Multivariable regression analyses of baseline characteristics associated with the composite of 2-year all-cause death, stroke, myocardial infarction and rehospitalization for heart failure. *AF, atrial fibrillation; BMI, body mass index; CABG, coronary artery bypass graft; CCS, canadian cardiovascular society; CI, confidence interval; COPD, chronic obstructive pulmonary disease; eGFR, estimated glomerular filtration rate; LVEF, left ventricular ejection fraction; MI, myocardial infarction; NA, not available; NYHA, New York Heart Association; OR, odds ratio; PAD, peripheric artery disease; PCI, percutaneous coronary intervention.*

Baseline characteristics	OR (95% CI)	p-val
Female	0.88 (0.67-1.16)	0.37
Age, year	1.02 (0.99-1.04)	0.14
Diabetes	1.28 (0.97-1.67)	0.08
PAD	0.98 (0.69-1.37)	0.89
COPD	1.52 (1.09-2.10)	0.01
eGFR, ml/min	0.98 (0.98-0.99)	<0.01
Prior CABG	1.07 (0.68-1.66)	0.75
Prior PCI	1.10 (0.84-1.46)	0.48
Prior MI	1.05 (0.74-1.46)	0.79
Prior stroke	1.52 (0.97-2.33)	0.06
Prior pacemaker	0.93 (0.58-1.44)	0.75
CCS > 1	1.10 (0.83-1.47)	0.50
NYHA > 2	1.27 (0.96-1.69)	0.10
AF	1.49 (1.12-1.97)	0.01
LVEF < 40 %	1.18 (0.83-1.66)	0.34

Supplementary table 9. Multivariable regression analyses of CAD characteristics associated with the composite of 2-year all-cause death, stroke, myocardial infarction and rehospitalization for heart failure. *CI, confidence interval; LM, Left Main; LAD, Left Anterior Descendent; LCx, Left circumflex; RCA, Right coronary artery; OR, odds ratio; PL, postero-lateral; PDA, Posterior descending artery.*

CAD characteristics	OR (95% CI)	p-val
LM	1.01 (0.65-1.54)	0.96
Proximal LAD	1.41 (1.06-1.87)	0.02
Mid LAD	0.79 (0.59-1.05)	0.10
Distal LAD	0.95 (0.58-1.51)	0.85
Diagonal	1.13 (0.79-1.61)	0.50
Proximal LCx	1.16 (0.82-1.63)	0.41
Mid LCx	1.28 (0.86-1.89)	0.21
Distal LCx/PDA	1.60 (0.92-2.69)	0.08
Obtuse marginal	1.49 (1.04-2.12)	0.03
Proximal RCA	0.87 (0.63-1.18)	0.36
Mid RCA	0.98 (0.70-1.36)	0.89
Distal RCA/PL/PDA	1.13 (0.76-1.64)	0.54
Calcific disease	0.83 (0.60-1.14)	0.25
Bifurcation	0.74 (0.53-1.02)	0.07
Multivessel CAD	1.06 (0.74-1.52)	0.74
